# “They don’t care about us”: older people’s experiences of primary healthcare in Cape Town, South Africa

**DOI:** 10.1186/s12877-019-1116-0

**Published:** 2019-04-04

**Authors:** Gabrielle Kelly, Lindeka Mrengqwa, Leon Geffen

**Affiliations:** Samson Institute for Ageing Research, University of Cape Town, 234 Upper Buitenkant St, Cape Town, 8001 South Africa

**Keywords:** Primary care services, Patient perceptions of care, South Africa, Older persons, Patient-centered communication

## Abstract

**Background:**

As older people age, they have different health needs compared to younger people. South African elder care policy places a strong emphasis on ageing in community rather than institutional settings, but the primary healthcare system is not geared to address the health needs of older people living in community settings.

**Methods:**

This paper presents findings of nine focus groups conducted with community-dwelling older adults in three areas (high, medium and low-income) in Cape Town, South Africa over 2 months in 2017. These discussions addressed primary health services available to older persons, their ability to access these services and their expectations and experiences of care.

**Results:**

Findings showed that while participants in the high-income area had few challenges accessing quality care or support services, services available in lower-income areas were much less responsive and participants displayed low trust in the healthcare system, feeling that their needs were overlooked. Participants who experienced poor doctor-patient communication often failed to comply with treatment, while those who experienced patient-centered communication, either through the private sector or NGO-public sector partnerships had better perceptions of care.

**Conclusions:**

Older persons’ complex health needs cannot be adequately addressed by a process-driven approach to care. Supporting patient-centered communication and care may help health workers to understand older persons health needs and improve patient understanding, trust and co-operation. This paper suggests the importance of community support services in enhancing health access and developing systems that enable healthcare providers to better understand and respond to older persons’ needs in resource-constrained settings.

## Background

Given the high costs of institutional elder care, many countries, including South Africa, are encouraging older people to remain in community settings for as long as possible [1, 2]. Building strong primary healthcare, home-based care and community-level social services, is crucial to maintaining the health and intrinsic capacity (the composite of all physical and mental capacities) of the community-dwelling elderly population and reducing frailty and institutionalization [3].

In most countries, health systems, and primary care systems in particular, are not well designed for older people, who have a different set of health needs to the younger population [4, 5]. Older people often have reduced physiological reserves and tend to have more complex and chronic multi-system problems, requiring more comprehensive and multi-disciplinary interventions that take the biopsychosocial aspects of health into account. This can present significant challenges to healthcare workers who often lack training in geriatric issues [3, 5–8].

This paper focuses on the experiences and perceptions that community-dwelling older adults have of primary care provision in Cape Town, South Africa. South Africa has the highest proportion of elderly people in its population in the African region (8.7%) and the number of elderly people (people over 60) is expected to increase from 4.1 million in 2011 to over 15 million in 2030 [9]. Health insurance coverage amongst the older population in South Africa is low (22.9%), particularly amongst the black (6%) and ‘coloured’^1^ populations (16.6%). These groups are much more reliant on the public health system or paying out-of-pocket for private care than the white elderly population, 73.5% of whom have health insurance [10]. Like all South Africans, older persons receive free public primary healthcare and beneficiaries who receive social assistance from the state in the form of a state pension (Old Age Grant), also receive free access to secondary and tertiary care. Yet, community-dwelling older people in South Africa face challenges accessing appropriate care and support and may under-utilise healthcare services or receive insufficient care [10, 11].

Healthcare access is shaped by both the individual characteristics of the patient such as financial position; social capital, which gives individuals access to information and networks; level of education and physical and cognitive capability, as well as supply-side factors in the healthcare system. Supply-side factors include: the availability of equipment, medicine and skilled human resources as well as facilities, policies, structures and processes [12, 13]. Although much work has been done in recent years to improve the availability and quality of health services, the South African healthcare system remains a “stressed institution”, mired by healthcare worker shortages, poor leadership and improper resource allocation. Studies of healthcare quality and responsiveness in the public health sector have shown that people of all ages face challenges in accessing adequate care in the public health system [13–16].

Older people, who have lower levels of functional capacity, face particular difficulties with transport to clinics, long waiting periods (which impose physical burden on older persons) and a general lack of health worker expertise on the management of chronic illness and geriatric issues [10, 11, 17]. However, those able to afford private healthcare, who are mostly white, report receiving more responsive and higher quality healthcare and having higher levels of self-rated health than those using the public healthcare system [11]. General Household Survey data also shows that people are much less likely to report problems with private care than public care [15].Census data also shows lower use of assistive devices and chronic illness medication amongst the African population compared with other wealthier population groups (particularly white people) in South Africa and higher levels of functional and cognitive limitation, indicating unmet health needs in this group [18].

These findings reflect broader patterns of income and race-related disparities in access to and quality of available healthcare services in South Africa, which affect the health, functional ability and quality of life of people in poor, predominantly African and coloured communities [18–20]. The biological ageing process is influenced by social, cultural, political and economic factors, with poorer elderly people having significantly lower health status, higher levels of frailty and disability and lower levels of well-being and quality of life than their wealthier counterparts [21–28].

Older people may struggle to access healthcare and are made more vulnerable to receiving inadequate care and having poor health outcomes by their diminished financial resources and intrinsic capacities, with those from disadvantaged social and economic backgrounds being particularly vulnerable. Despite the policy move towards community-based care, there are no special services or programmes offered to older persons at the primary care level and they must compete with other patients for care [7, 29]. Given time and resource constraints, fee structures and the typical nature of doctor-patient interactions, many public healthcare professionals do not provide older people with adequate care or struggle to identify frailty and dementia [34–36. Services tend to be clinic-based and focused on acute conditions and healthcare workers often lack the training to deal with the specific needs of older people. There is limited training in the medical or nursing curricula at both undergraduate and postgraduate levels [7, 30]. The complex needs of older persons can result in negative attitudes by healthcare workers, which can affect care [31].

As a result, health issues affecting the aged are often undiagnosed or overlooked in primary care settings [32–34]. Although few studies have been conducted, there is general agreement in the literature that access to care and health systems responsiveness in developing countries is poor, and that health systems fail to meet the needs of older people [34, 35]. Studies of healthcare use and responsiveness amongst older persons [11, 35] and other vulnerable groups [36] in South Africa indicate high levels of dissatisfaction, low levels of care quality and a lack of trust in healthcare professionals in both rural and urban settings. Challenges in accessing public primary health services will only grow as the population ages, the burden of chronic disease, frailty and disability increases [29] and demand for chronic care within primary healthcare, as well as long-term care and community support services grows [7]. This is compounded by the growing burden of chronic communicable and non-communicable disease on the health system across age groups [12]. Despite this growing challenge, there has been little study of existing care gaps for the elderly in the country and little recent research on older people’s perception of health and use or experiences of healthcare services in South Africa [5]. Of the studies that do exist, few are focused on community-dwelling adults, which comprise the majority of older persons [11, 24, 35, 37]**.**

In summary, from the existing literature it is clear that more attention needs to be paid to the health needs of older persons and the extent to which they are being met by both the public and private primary healthcare system in South Africa. This paper contributes to building much-needed evidence in this area.

## Methods

### Methodological approach

The aim of the study was to understand older persons’ experiences of primary healthcare services in their communities. The data presented forms the first part of a broader study aimed at understanding people’s experiences of ageing in these communities. To achieve this goal and allow the ‘voice’ of older persons to be heard in the study, an inductive and exploratory approach was taken to the research *based on* the qualitative methodology of *grounded theory* [38, 39] using Charmaz’s constructivist approach [40].

Grounded theory (GT) uses the methods of coding and constant comparison of emerging findings to discover relevant concepts, categories and relationships between them through a theoretical framework that emerges from the research itself through a process of induction [38]. We used elements of this approach but did not aim to generate new theory as such. For practical purposes and because of ethical review board constraints, we also did not employ the GT principal of theoretical sampling.

Focus groups (FGs) were chosen as a data collection method because a group dynamic provides opportunities for participants to set the agenda and priority is given to their understanding of the world [41]. Focus groups also offer opportunities to gather a multiplicity of views and observe participants engaging in interaction that is concentrated on attitudes and experiences. [42]

### Research setting

The research was conducted in the Cape Town Metropole region in the Western Cape Province, in three different communities selected to maximise variation in terms of economic status and demographic composition, as well as access to health and social services and social and cultural factors. The three sites included are: Khayelitsha, a low-income area occupied by mostly black isiXhosa speakers; Lotus River, a predominantly ‘coloured’ area occupied by low to middle-income Afrikaans speakers; and Sea Point, a high-income area populated largely by white English speakers. These research sites are a good representation of the population in Cape Town, which is dominated by isiXhosa, English and Afrikaans speakers and is highly geographically divided by race and income. In Lotus River and Khayelitsha, the majority of participants were recipients of Older Persons Grants – a monthly pension of R1690 ($117), on which other family members were often also reliant in poor households (Table 1).

**Table 1:** Key population statistics in study sites (Source: Statistics South Africa, 2011)

Key Statistics	Sea Point	Khayelitsha	Lotus River
Total population	13,332	391,749	38,143
Elderly (65+)	10.1%	1.6%	7.2%
Dependency ratio	39.8	42.5	48.3
Higher education aged 20+	37%	4.9%	10.1%
Completed high school aged 20+	30.8%	30.8%	29.2%
Formal dwellings	96.6%	44.6%	92.3%
Flush toilet connected to sewerage	97.1%	71.7%	93.2%
Piped water inside dwelling	96.9%	34.6%	92.8%
Electricity for lighting	99.3%	80.8%	97.8%
Main language spoken	English (68.8%)	isiXhosa (90.5%)	English (53.7%); Afrikaans (44%)
Main racial group	White (67.5%)	Black African (98.6%)	Coloured (92.6%)
% of Households with incomes of R4800 or less	10.7%	24.5%	10.8%
Unemployment rate	9.5%	38.3%	15.1%

### Recruitment of participants

Participants were recruited with the help of four Cape Town-based non-governmental organisations (NGOs), which provide health and wellness, social and housing services to elderly people in the area. With the exception of one focus group in Khayelitsha, all participants in this study were members of seniors’ clubs run by these NGOs, who pay a small monthly fee to participate in club activities. In Sea Point and Lotus River, three focus groups were run at each seniors’ club. In Khayelitsha, participants were drawn from three different clubs – two seniors’ clubs affiliated with two different NGOs, as well as an independent seniors club run by a group of elderly people without funding or support. The first group was affiliated with an NGO that runs its own clinic on site (NGO 1). The second group was associated with an NGO that provides healthcare support services to facilitate access to better care in community clinics (NGO 2). The third independent club provided no services to members except a place to socialise. The purpose of including three different groups in Khayelitsha was to have variety in terms of the kinds of healthcare services received. These services are not offered by NGOs in Lotus River or Sea Point, where levels of poverty are much lower and access to services are better.

Within each of the neighbourhoods, focus group participants were purposively selected to ensure diversity in the sample in terms of living arrangements, age, gender and background. Sixty-four participants - 52 females and 12 males, participated in the study. The low number of males reflects overall membership patterns of the seniors’ clubs involved. Twenty-four participants lived in Lotus River and surrounds (Retreat, Grassy Park and Wynberg), 16 lived in Sea Point and 24 lived in Khayelitsha. All participants lived in the community and self-reported function and cognition was intact and all remained sufficiently independent to participate in club activities (Table 2).

**Table 2:** Description of participants

Age range	59–92
Mean age	74.1
Number of participants Lotus River (3 FGs)	24
*FG 1*	*9 (7 women; 2 men)*
*FG 2*	*8 (8 women)*
*FG 3*	*7 (5 women, 2 men)*
*Race of participants*	Coloured
*Home language of participants*	Afrikaans / English
*Health system use*	100% of participants used public primary health clinics for routine care (some reported occasional use of private GPs for acute issues) 1 (hospital coverage only)
*Number of people with health insurance*	
Number of participants Sea Point	16
*FG 1*	*5 (3 women, 2 men)*
*FG 2*	*6 (6 women)*
*FG 3*	*5 (5 women)*
*Race of participants*	White
*Home language of participants*	English
*Health system use*	100% used private healthcare providers for primary care
*Number of people with health insurance*	16
Number of participants Khayelitsha	24
*NGO 1 (1 FG)*	*9 (8 female, 1 man)*
*NGO 2 (1 FG)*	*6 (6 females)*
*Independent seniors’ club (1 FG)*	*9 (4 women, 5 males)*
*Race of participants*	Black African
*Home language of participants*	isiXhosa
Health system use	62% of participants used public primary health clinics for chronic and acute care. 38% used NGO 1’s clinic.
Functional capacity – use mobility device	9
Functional capacity – fully independent	54
Functional capacity – need full-time care / support	1
Living arrangements - alone	16
Living arrangements – with family	38
Living arrangements – in retirement village	6

### Data collection

Three focus groups were run in each area (total of 9 groups) which varied in size from 5 to 9 members and lasted 60–90 min. About half of this time was spent discussing older persons experiences of the health system, although this varied between groups. Focus groups were conducted by the first two authors, with one acting as facilitator and the other co-facilitator for each group. The focus groups were guided by a set of broad questions aimed at promoting discussion about their health needs between participants. Questions related to the healthcare experiences of care of participants are included in Table 3. Additional probes were developed in successive focus groups to flesh out emerging categories.

**Table 3:** Focus group guide questions on access to and experiences of health services

Who in the community provides healthcare services to older people?	
Why and when do you visit this person/facility?	
What do you expect when you visit?	
What is it like when you visit there?	
How are you treated by health care staff?	
How do you get to the facility? • How easy is it for you to get there?	
Do you feel that your health needs are addressed	
If you have visited both a public and private facility, have you noticed any differences in the care that you received?	

The focus groups’ discussions were facilitated in the home language of the participants (English, isiXhosa or Afrikaans) to ensure that everyone had an equal opportunity to participate and express their opinions and feelings freely. All focus group discussions were recorded, translated and transcribed. To ensure the accuracy of translation, translations were carried out by the focus group facilitator and checked by the co-facilitator, who was also present during the focus group and therefore aware of the conversational content and context. Field notes were taken by both the facilitator and co-facilitator during the focus groups and additional thoughts and experiences of interacting with participants were made directly after the focus groups. All the focus groups were conducted in the facilities belonging to the organisations mentioned above because the surroundings were familiar and convenient to the participants.

### Data analysis

The central concept of data analysis in GT is *coding,* which is a process of separating, sorting, distilling and synthesising data by firstly breaking it into segments, coding these segments by allocating labels to them and then comparing codes and data, building the codes into categories and making analytical connections between categories that allow theory to emerge [40]. All data, including the field notes of the two main authors, was coded and analysed using NVivo Version 11.4.0. Data was first coded using open coding and was then re-coded into categories and then themes.

The research team held a data analysis workshop to discuss coding and their interpretations of data. The first and second author, who did the majority of the data analysis, come from different cultural and language backgrounds, providing opportunities for rich discussion around interpreting the data. It is important to engage in reflexive practice to improve the credibility of qualitative research and reduce confirmation [43]. To achieve this, the facilitator and co-facilitator compared and discussed their field notes after every focus group. Referring back to these notes during the analysis ensured that the findings remained grounded in the data.

Results and a provisional analysis of the results were presented to community organisations involved in recruiting research participants for their comments and input. This was carried out both for the purpose of participant validation and our ethical obligation to disseminate research to stakeholders for advocacy and community and clinical practice. Findings were also shared with participants via Powerpoint presentations to each group. Research participants affirmed that the interpretations of findings reflected their feelings and experiences. NGO staff, as well as an older persons’ sector task team from various other organisations, indicated that results supported their experiences of working with older persons.

### Ethical approval and consent to participate

The research protocol of the study was approved by the Faculty of Health Sciences Human Research Ethics Committee at the University of Cape Town. All participants gave informed written consent and the anonymity and confidentiality of the research of all participants was maintained throughout the research process. Participants were, however, advised that confidentiality could not be totally guaranteed in a focus group setting. All consent forms indicated that excerpts of the focus groups would be included in any publications emerging from the research. Other than indicating whether the person was an older man or older woman (all participants were older persons), no information is included in published data that would allow other club members, staff of the older persons clubs or the general public to identify participants.

To thank them for their time, all study participants were given a R50 voucher after the focus group discussion. To avoid pressurising people to participate, potential participants were not made aware of the voucher until the end of the focus group.

## Results

### Summary of findings

While cost and physical access were not a challenge in obtaining care, participants in Lotus River and Khayelitsha using public healthcare faced significant challenges in accessing quality and age-friendly care. Participants in these focus groups had low levels of satisfaction with care and had strongly-held perceptions that their health needs were overlooked. Challenges experienced by these participants included long waiting times and the lack of prioritisation of older people, the negative and unhelpful attitudes of healthcare staff, shortages of medical personnel, rushed consultations and lack of examination by doctors, poor continuity of care, lack of patient education and, in a few cases, non-availability of medication. Members of seniors’ clubs Participants in Sea Point did not experience these challenges. However, they did not necessarily receive patient-centered or age appropriate care from busy private practitioners who rarely or never addressed issues related to ageing or function.

### Health service access and utilisation

With the exception of two participants, who claimed to avoid doctors, all participants received primary healthcare services for chronic conditions, acute problems or regular checkups. Participants in Sea Point all had health insurance and exclusively used private providers, while participants in Lotus River and Khayelitsha predominantly used public health services out of financial necessity.

Primary health services are easily and freely available through clinics or community health centres (often referred to as day hospitals) within most urban communities in the Western Cape and physical access and cost of services was therefore not a problem to the majority of participants, who were still relatively mobile. However, those who could not walk or get public transport and had no family member with a vehicle, had to hire transport to get to the clinic or a hospital they were referred to, presenting an often considerable cost to accessing care. There is a government subsidised Dial-a-ride transport service available to people with disabilities, but participants were not aware of how to register for and access this service, as one woman described below:

*“I have to hire transport when I have to attend the club for a doctor. I don’t know what to do. I see the cars that take disabled people around, but I don’t know how to get that service.”* (Older Woman, Khayelitsha, Independent Seniors’ Club FG)

Community health workers do deliver medication to frail older people living in communities, but this service was not available to most participants in this study who were still relatively mobile and active and only one woman had her medication delivered to her home.

Specialist care was not so easily available to those using public health services, with long waiting lists being the primary barrier to access.

*I know a lady who had to wait 2 years for the doctor. I saw that same doctor in a week - I got an appointment a week later. I went to him and everything is sorted out but she waited 2 years because she didn’t have medical aid. (Older Woman, Sea Point FG2).*

In Lotus River, many of the participants had health insurance during their working lives, but with their greatly reduced retirement incomes or Old Age Pension, could no longer afford it and had entered the public sector for routine care. None of the Khayelitsha participants had ever been on health insurance.

Two of the Khayelitsha groups and the three Lotus River groups attended the chronic health clubs at their clinic, where they collected their medication free-of- charge and received a six-monthly routine check-up from the doctor or clinic sister. They also used the primary clinics for acute health problems, but in some cases chose to rather pay for private services to avoid long waits at their community clinic.

This mixed use of public and private care is fairly common in South Africa and in 2016, the General Household Survey estimated that over 1.5 million households without health insurance used the private healthcare sector [37]. None of the participants reported using traditional or alternative medicine or practitioners as an alternative to mainstream medical care.

Participants in Sea Point, who exclusively used private care, had no such barriers to access, with some practitioners even conducting home medical visits.

*“We live in a very privileged society…we’ve got a choice of doctors and we’ve got Clicks and Dischem [pharmacies] at a reasonable price with all these tests [health screening]. We live in a beautiful bubble.” (Older woman, Sea Point, FG 2)*

### Waiting for services

The South African public healthcare system is characterised by long waiting times [36]. For participants, waiting for long periods to see a healthcare professional or at the pharmacy, was an expected part of the process of seeking care:

*Participant 1: It’s the day hospital. You must wait.*

*Participant 2: That’s why they call it the day hospital. You sit. You spend the day there [Laughter]. Even to collect medication*

*Participant 1: You stay there waiting for the medication then after waiting all that time they come and tell you that your medication is not there*

*Participant 2: Mmh…you wait there for your medication until the sun sets - you don’t wait because there are no workers at the clinic*

*Participant 1: They are there*

*Participant 3: There are workers there chatting about their own things telling each other their weekend stories while we wait and nothing moves*. (Lotus River FG3)

Due to mobility issues, older people who did not have children with cars and used public transport or walked to the clinic, found it difficult to get to the clinic as early as younger people and struggled with the physical challenges of spending hours waiting in line.

*“If you don’t have money you will be in pain, because we don’t have cars.” (Khayelitsha FG1)*

According to Western Cape Department of Health policy, older people, children and people with disabilities should receive preferential treatment [36] in health facilities, but this did not appear to be the case in the clinics frequented by participants, except in one case where a participant with a visible disability was served earlier than others.

*“Yes, these ladies have said it all … we as old people are not treated well that is something we must admit we cannot run away from that…we spend long hours at clinics …they usually say or even put notices on the walls at clinics indicating that the procedure is to start with children and then older people … that is not the procedure that is followed.” (Older Woman, Khayelitsha, NGO FG1)*

Lotus River participants, who were served by clinics in Grassy Park, Retreat, Lotus River and Wynberg, reported that the introduction of chronic disease ‘clubs’ had greatly improved the speed of service delivery. Chronic clubs serve clinically stable patients with chronic diseases of lifestyle who are seen separately from other patients every 6 months at specific appointment times, making it quicker to receive routine care and collect medication, which they received at their appointment, rather than monthly. Clubs should also offer healthcare education, community support groups, nutrition services, general health observation and routine blood tests and home-based care referrals and retinal screenings [44]. However, based on participants’ descriptions, it seemed that services such as health education, nutrition services and community support groups were not always available. In Khayelitsha, it was reported that long queues to collect medication remained a problem and six-month follow-up appointments often took the whole day. Participants at both sites also complained that chronic club patients can only be seen for their specific diagnosed condition (e.g. diabetes or hypertension) and had to use regular clinic services if presenting with any other complaint, requiring them to spend many hours waiting for service.

These waiting periods are largely a function of high demand for services and staff shortages (particularly of doctors) or absenteeism and are well-documented in other studies [45, 46]. According to participants, this problem was compounded by a visible lack of interest and motivation on the part of nurses, whom every group complained spent significant periods of time socialising rather than dealing with patients:

*“The staff goes up and down, but never take the folders and do their jobs. Sometimes I feel sorry for people that are clearly in need of urgent attention but those ladies there are in no rush, they take their time. No one cares about us at the clinic.”* (Older Woman, Khayelitsha, NGO FG2)

Participants felt that being kept waiting was seen as a sign of disrespect and lack of interest in them as individuals. In one Khayelitsha group participants repeatedly declared: “they don’t care about us”. In contrast, there were much shorter waiting times at the offices of private GPs, which was the main driver of private healthcare use amongst those without health insurance plans.

### Perceptions of treatment by healthcare workers

#### Attitudes of healthcare staff

Demand for services and the shortage of healthcare workers has created an over-stretched and de-motivated workforce, resulting in high levels of burnout and inadequate service provision [46–50]. Participants attending clubs Khayelitsha and Lotus river complained extensively about the attitudes of nursing staff towards patients, which was characterised by disinterest, value judgements, rudeness and even aggression if the patient was perceived to be behaving incorrectly (for instance, missing an appointment).

*“We are afraid of them healthcare workers; these children are like dogs when they catch you. You will as if you’re in a corner being bitten by dogs when they start shouting at you.”* (Older Woman, Khayelitsha, NGO FG 2)

*“Our children treat us badly at these clinics, they don’t care. We see them chatting over there until they see a doctor - then they start working.”* (Older Woman, Khayelitsha, Independent Seniors’ Club FG)

As a result of this behaviour, nurses have a negative reputation in many South African communities and patients may be less likely to seek care or adhere to treatment because of being treated harshly or with disrespect [14, 51, 52].

Clients seen by frontline service workers as ‘weak’ are most likely to receive poor treatment from frontline service workers and this can exacerbate the exclusion and impoverishment of vulnerable groups such as older persons [53].

Being afraid to ask questions made it difficult for older people to understand and navigate clinic systems and processes, which often changed between visits. Although opportunities exist to complain to clinic managers about poor treatment or submit a complaint via the complaints box, participants saw these mechanisms as largely ineffective. They also feared that complaining directly to healthcare staff could result in negative or even punitive reactions from nursing staff, such as placing the patient’s file at the bottom of the pile to delay treatment, which some had experienced or witnessed happening

*Participant 1: If you complain they take advantage of that and then you know what they do? They make you wait longer.*

*Participant 2: Yes, you can’t complain. They take your file and they put it right at the back*

*Participant 1: So you are not allowed to say anything.* (Lotus River, FG3)

Participants connected the negative attitudes of healthcare workers to what they saw as broader patterns of ageism and lack of respect and care for the elderly in their communities, where they reported that high levels of elder abuse and neglect exist and their voices were not taken seriously; claims that are well-supported by the literature [54].

*“I want to be valued because we are not valued as we grow older even in clinics. You find that the nurses dismiss everything we say, they say ‘no don’t mind them, they talk too much nonsense.’ You find that when we go to the clinic no one pays attention to us as older people. Even when we go to places like banks - FNB for example - they never say here is a chair for an older person or older people should go first and not stand on the lengthy line. We are not valued because people are just waiting for us to die already because you are useless. You don’t add any value to anything in life.”* (Older Woman, Khayelitsha, Independent Seniors’ Club FG)

### Relationships between healthcare workers and patients

While participants in Sea Point had long-standing and trusting relationships with specific general practitioners, whom some could call on their cell phones if necessary, the public health system was marked by a lack of continuity of care and the absence of interpersonal relationship between doctor and patient.

*“My GP, I can phone him at 7/7:30 in the morning when I’ve got some silly question to ask and he answers me, he helps me. I don’t have to have any other GP.” (Older woman, Sea Point, FG 1)*

*“They change doctors every day, this month it’s this doctor, next month it’s another one. These doctors can’t keep track of your health because they get changed so many times.”* (Older Woman, Khayelitsha, Independent Seniors’ Club FG)

Despite receiving better-quality care than other groups in the study, numerous participants from the Sea Point focus groups complained that medical practice had changed, becoming less patient-centered, more rushed and increasingly impersonal over time.

*“Today I feel doctors are not like the old doctors we used to have they’re not really caring about you anymore it’s about money.” (Older Woman, Sea Point, FG 3)*

Participants attending chronic clubs were more often seen by nurses than doctors for their routine six-month checkups, which was disappointing to participants who expected to see a doctor and had low trust in nurses. Even when participants were able to see doctors, they often remained dissatisfied, complaining about the rushed nature of consultation and the lack of examination, communication and education about their conditions, medication and side-effects they experienced.

### Lack of physical examination

The lack of physical examination was of particular concern to participants. Although aware of the shortage of doctors in clinics, participants described how not being examined made them feel unimportant and not worth the time or energy of healthcare providers:

*“They carry their equipment around, but they never use it to examine you …you rarely find a doctor that examines you.”* (Older Woman, Khayelitsha, NGO FG 2)

*“When you are with the doctor, the doctor asks you questions. They never touch you. You just tell them maybe your back is sore, but they don’t examine you. The nurses check the high blood pressure and urine sample, but if you get there and tell them that you are sick with this they just write it down and tell you to go to the pharmacy.”* (Older Woman, Khayelitsha, NGO FG 2)

*“He doesn’t even have a stethoscope on him to check your… We’re OLD, so without our knowledge something can be wrong. I mean we can have a murmur on our heart, anything…they don’t touch you. No, they just look at you and say to you “oh, okay sore chest, cough for me” but they don’t have no stethoscope. Like if you go to a private doctor he will check your throat and put that spatula into your tongue. “Open your mouth, cough…” all those things. They examine you. But when you go there [day hospital] they just give you the medication… That’s one of the biggest gripes I have, because what are we doing there? Why must we see doc? And they tell you that you must see doctor every six months!”* (Older Woman, Lotus River FG 3)

Although doctors had the patients’ file available and nurses usually captured data such as weight and blood pressure prior to visits with the doctor, participants felt that the lack of examination was likely to result in misdiagnosis or incorrect treatment and was seen as a sign of incompetence as well as lack of care. As one participant noted, “They make mistakes and they are negligent.” (Older Man, Lotus River, FG3). Participants in Lotus River and Khayelitsha also felt that medicine had ‘changed’ in this regard and felt that older doctors were more likely to examine them than younger doctors.

Other studies have shown that due to time constraints, primary care doctors often fail to physically examine patients [55, 56]. Participants from all groups expressed numerous times that as they aged, retaining their health was extremely important and the key to retaining their independence and quality of life. It was therefore very concerning to them that important health problems may be overlooked by inattentive doctors.

However, in some cases, there was discordance between participants’ expectations of services and investigations and what is realistic and appropriate. For example, some participants wanted x-rays for diagnostic purposes when they were perceived as unnecessary by doctors, or expected electrocardiograms (ECGs) or blood tests as part of general health checkups, seeing their inability to access these services as denial of care.

### Routinisation of care

Medical care to participants was not patient-centered and was highly routinised. Participants expected doctors or nurses to explain their medical conditions, medication and potential side effects or adverse reactions to them, but this rarely happened. A number of participants felt that instead of listening to their problems or proactively identifying ways to improve their quality of life, their conditions were quickly dismissed as the complaints of old-age and they were simply prescribed more medication for their diagnosed problem.

*Participant: You go to the clinic with your own illness like this arthritis, they don’t pay attention to what you tell them. Like now winter is coming we have aches, I have something under my foot it’s like a corn, it makes it difficult to walk. I’ve reported this at the clinic several times, but no help is given to me*

*Interviewer: How do they respond when you tell them?*

*Participant: They write down and they just give me my medication for high blood and arthritis because that’s what I’ve been diagnosed with.* (NGO FG 2, Khayelitsha)

Participants felt that doctors displayed very limited interest and empathy and described having their questions, requests or complaints brushed off and there was a strong perception that they were not seen as valuable because they were old or that their health issues were just inevitable parts of old age that they would have to deal with and offered no support or advice in this regard.

*“As older people, sometimes when you say something, it seems as if you are crazy and no one takes you seriously.”* (Older Woman, Khayelitsha, Independent Seniors’ Club FG)

*“But there is something about this now and the other ladies can bear me out…the elderly is not getting the same attention as the young people. They also refuse to give you an op or something because they feel…the hospitals have also got that attitude now. “You are too old” So the young person will get seen to because he’s still going to be a benefit or whatever else but we as the elderly we are not going to get that privilege.”* (Older Woman, Lotus River FG 2)

A number of participants argued that once they were categorised as a chronic case, their treatment became highly standardised. When prescribing medication, it appears that not a lot of attention was paid to what medications patients were already receiving, leading several participants to complain about the mix of medications they were prescribed. Participants complained on numerous occasions of going home with large bags of pills which they did not understand the purpose of and often did not use. Several of them had stopped taking some or all of their medication because it made them feel unwell.

*“They give you a bunch of pills…they ask you what is wrong. You say: ‘I have a pain’ and then they prescribe the pills for to you. ‘I have that pain’ and they write up more pills. In the end you leave the day hospital with a bag of pills like this [indicates big bag]. Now I begin to take them as they prescribed and then the whole room started spinning. Then I told them and they said ‘no, that’s the beginning of the pills, you must take them’ and I said ‘no more!’. I am never going to take all those pills again because I can see that those pills make me sick. Because they didn’t investigate me, they just asked me what bothered me, and I got pain pills and she wrote up this bag of pills and they gave it to me. They don’t investigate.”* (Older Man, Lotus River FG3)

*“You find signs of this even with doctors you find that you’re 60 years old, but they give you so many pills, pills for all sorts of things with no care of following up on how the pills affect you…”* (Older Woman, Khayelitsha, NGO FG 2)

*“I never took the urine tablets [diuretics] because I was like ‘why is he giving me this?’”* (Older Woman, Lotus River FG2)

Overall, participants felt they were being provided with inferior service in the public sector, which they were expected to accept because it was free. They were upset by the perceived need to give up one’s agency or right to dignity when they enter the public healthcare setting. In one focus group in Khayelitsha, participants attributed poor service standards to the fact that patients were poor and black and had no other options.

### Experiences of private care

Those that had experienced private healthcare had far fewer complaints about these services and felt doctors and other healthcare staff in these settings were attentive and thoroughly examined patients, resulting in better-quality care.

*Participant: Just before Christmas I fell and had a gash in my head. I don’t know why I fell, but I went to a private doctor and they examined me very well.*

*Interviewer: Is it different from how they treat you at the day hospital?*

*Participant: It is completely different.*

*Interviewer: So how is it different?*

*Participant: The way they treat you. And you can talk to him – you can tell him this and that. He has the time to see to you.* (Older Man, Lotus River, FG1).

However, it appeared that there was still little time during private consultations to focus on health promotion or issues around function or wellbeing in relation to ageing.

*Participant 1: He explains in a way that I have to ask my son explain again what he’s saying…*

*Participant 2: You probably need someone with more of a bedside manner.*

*Participant 1: Yes, because I need someone to explain to me what’s happening to my body, but he has no time for that. (Older Woman, Sea Point FG 2).*

*Participant 1: They treat you with respect but they’re busy.*

*Participant 2: They don’t give you the time that they used to.*

*Participant 1: Unless you volunteer information, they don’t ask…They don’t have time because the waiting room is full and they’re fully booked. They do what they need to do. They don’t have time to be your psychologist. (Older Woman, Sea Point FG3).*

The cost of private care and medication was seen as problematic and doctors were perceived as greedy for charging such high consultation rates for such appointments. Even participants in Sea Point, who all had health insurance, found that the costs of private care were often not fully covered by their schemes or that they maxed-out their coverage before the end of the year.

This left participants from across all groups cynical and wary about both the public and private healthcare system, particularly those in Lotus River and Khayelitsha who had very low levels of trust in any healthcare professional.

### The role of seniors’ clubs in facilitating access to services

Findings showed that being a member of a seniors’ club improved access to health services and perceptions of receiving quality care. Members of Khayelitsha NGO 1 paid an additional fee on top of their seniors’ club fee to be a part of a clinic run through the NGO as part of a public-private partnership. This allowed them to pick up their monthly medication from the state at the NGO clinic and see the clinic doctor every 6 months, as well as access services like dementia support. Members of this club participating in the study indicated that they were prepared to pay this fee primarily to avoid what they felt was undignified treatment in free public clinics. As one woman noted, “I became a lady ever since they opened this clinic”. They also benefitted from having an established relationship with the nurse and doctor who worked at the clinic, trusting that their health was being monitored and feeling valued as an individual because of this relationship and the time spent examining and speaking with patients.

*“Here [NGO 1 clinic] I am being treated nicely, having a nurse that is friendly and caring. I don’t spend too much time waiting at the clinic the nurse calls me...then after she sends me to the doctor. I only receive such treatment now, because all these years before I was attending the day hospital.” (Older Women, Khayelitsha, NGO 1 FG)*

The seniors’ club also provides daily transport to members, making physical access to the clinic easier for those with mobility restrictions. The other NGO provided transport to the clinic for appointments, health workers to deliver medication and assistance with welfare applications and home visits to those who are frail and home-bound, provided health monitoring and offered participation in exercise and wellness programmes.

*“We get social workers here, we get our medication here. You must give them your prescription so you don’t have to go to the clinic – you only go to the clinic when it’s the date for your club at the clinic…The glasses I have, I got them when optometrists visited the club” (Older Woman, Khayelitsha, NGO 2 FG)*

## Discussion

While access to primary healthcare has improved in post-Apartheid South Africa [20], and chronic clubs and other administrative interventions have somewhat improved care for many older people, these efficiencies have not led to adequate and acceptable services from older users’ perspectives [36].

Results from this study have shown that, particularly in the public sector, older persons are dissatisfied with the quality of healthcare available to them and feel that their needs as older people are particularly neglected. Findings also demonstrate the stark income-related disparities that exist in terms of access to and quality of care. These findings support and build on the survey results of the World Health Organisation’s (WHO) *Study on Global AGEing and Adult Health* (SAGE), which evaluated the perceptions that participants aged 50 and older had of health system responsiveness in South Africa. The study found that, even in the private sector, healthcare responsiveness in South Africa was much lower than in other developing countries included in the study [11]. The Western Cape, where the study took place, has more than four times more doctors than in other province, indicating that challenges are likely even more pressing elsewhere in the country [57].

Findings showed that due to poor communication, participants were confused by health system processes and lacked information on or misunderstood their diagnoses and treatment protocols, resulting in poor compliance. Implementing a more patient-centered approach to patient care, which addresses patients’ needs, feelings, fears and expectations, may help to align patient expectations with evidence-based protocols for care.

Patient-centered care is defined by the Institute of Medicine as care that “is respectful of, and responsive to, individual patient preferences, needs and values, and ensuring that patient values guide all clinical decisions.” [58] Patient-centeredness is now understood to be one of the central components of quality care and has been shown to improve patient knowledge, self-efficacy, adherence to recommended treatment, and self-management of chronic disease which can improve health outcomes, improved quality of life and well-being and reduced care costs and greater equity in distribution of health across populations [58–62]. Patient-centered care involves treating the whole person in their social context, establishing a partnership that involves listening to patients needs and preferences, trust, emotional support and patient education and enablement [59] – things which were clearly not present in the care received by participants in this study.

In their study of nurses’ experiences of the implementation of free primary healthcare, Walker and Gilson found that nurses who felt overworked, frustrated and isolated in an under-resourced healthcare system coped with their work environments by blaming patients and categorising them into those deserving or undeserving of care [63].

Other studies have shown that healthcare providers employ similar coping strategies that detached and distanced themselves from the needs of clients and patients and could lead to a lack of professionalism, indifference and neglect [64, 65]. This does not mean that people were docile in their receipt of care and there were some participants who had been able to navigate better access to the healthcare system. For example, by calling the head nurse to complain that the doctor had not examined her, one woman in Khayelitsha was eventually examined by the doctor.

The lack of patient-centeredness, poor communication and the lack of prompt attention have resulted in a lack of trust in the healthcare system and professionals. A lack of trust and time costs associated with seeking care can lead to reduced healthcare utilisation [35] and as this study has shown, compliance with treatment. This may make it more difficult to support the health and well-being of older persons in community settings. Although they can be difficult to implement, more holistic and patient-centered models of care have shown positive results in terms of patient trust and increased sense of responsibility for their own health [66, 67] .Improving the quality of interactions between clinicians and patients through patient-centered communication [60] would also help to reduce patient intimidation caused by fear of medical authority and imbalances in power relations, which can act as a barrier to effective communication and make it difficult for patients to exercise agency in healthcare interaction [56].

As well as pointing out the failures of the healthcare system, this study has also shown the importance of NGO support in improving elder care quality and the dignity of care, which participants considered very important. In all three communities, older persons derived significant benefits from participating in NGO activities and receiving health and social services. Whilst in Sea Point this was limited mainly to socialising, a stark contrast could be seen between those receiving health and social services and support from the two NGOs in Khayelitsha and those who were not.

### Limitations of study

Since the participants regularly attended seniors’ clubs, the sample does not include any community-dwelling adults with significant functional or cognitive limitations who are home-bound and are particularly vulnerable. Most had access to NGO services and may have been better-off than those who do not have easy access to social or health workers. The inclusion of the independent seniors’ club members in Khayelitsha did, however, allow us some insight into the experiences of those who don’t have access to these services. Although male participants did not air substantially different views from the women present, the low number of males participating in the study may be seen as a limitation in our understanding of older men’s perspectives on health access.

While focus groups were useful in gathering a variety of perspectives and generating debate, there is obviously less confidentiality possible in a focus group than in individual interviews. While people were open and told personal stories in most of the groups, in some there was initial awkwardness or hesitancy and, in one group, this was difficult to overcome.

## Conclusions

For older people to remain functional and active participants in their communities, their specific health needs need to be considered by the healthcare system. This study has shown that the healthcare system in Cape Town (and likely the rest of the country) underserves older persons, who have negative perceptions of the care they receive. Care is heavily focused on ‘processing’ patients and patient’s individual needs and concerns are overlooked, leading to non-compliance with treatment.

Findings showed that more patient-centered care, as provided in private settings or NGO-public sector arrangements, result in perceptions of better care. As time constraints seem to be a major obstacle to patient-centered communication, there is a need to increase the number of healthcare workers in the public sector to address demand-side challenges to care that affect all population groups [57], and older persons in particular. As human resources shortages have been a long-standing challenge, making healthcare more patient-centered and age friendly will also require novel and innovative models to improve the wellbeing of older persons. More research is needed on the role that community initiatives and task-shifting to community health workers can play in strengthening health service delivery. For instance, by developing appropriate information systems, geriatric screening performed at the community-level could help doctors to better understand and respond to the complex needs of older persons during short primary care consultations.

Furthermore, there is a need to train healthcare workers on issues related to geriatrics and gerontology so that they are better-able to recognise the needs of and provide appropriate care to older persons. Ageist attitudes have negative consequences for care and need to be addressed.

## Abbreviations

FG: Focus group
GT: Grounded theory
NGO: Non-governmental organisation

## Footnotes

1 In South Africa, the term ‘coloured’ refers to the ethnic and cultural group made of persons of mixed-race, Khoi or Asian descent. The term emerged as a result of apartheid classification system, but is still commonly used today and is not considered to be pejorative. ‘Cape Coloureds’ are the largest population group in the Western Cape, the province where the study was carried out.

## References

[1] Lette M, Baan CA, van den Berg M, de Bruin SR. Initiatives on early detection and intervention to proactively identify health and social problems in older people: experiences from the Netherlands. BMC Geriatr. 2015;15. 10.1186/s12877-015-0131-z. 10.1186/s12877-015-0131-z PMC4628317 26518369

[2] Rahmawati R, Bajorek B (2015). A community health worker-based program for elderly people with hypertension in Indonesia: a qualitative study, 2013. Prev Chronic Dis.

[3] World Health Organization, editor. World report on ageing and health. Geneva, Switzerland: World Health Organization; 2015.

[4] Banerjee S (2015). Multimorbidity—older adults need health care that can count past one. Lancet.

[5] Aboderin IAG, Beard JR (2015). Older people’s health in sub-Saharan Africa. Lancet.

[6] Hoogendijk EO, Muntinga ME, van Leeuwen KM, van der Horst HE, Deeg DJH, Frijters DHM (2014). Self-perceived met and unmet care needs of frail older adults in primary care. Arch Gerontol Geriatr.

[7] Kalula SZ (2011). The quality of health care for older persons in South Africa: is there quality care?: conference paper. ESR Rev Econ Soc Rights South Afr.

[8] Stuck AE, Iliffe S (2011). Comprehensive geriatric assessment for older adults. BMJ..

[9] United Nations Department of Economic and Social Affairs, Population Division. World Population Prospects: The 2015 Revision. United Nations Department of Economic and Social Affairs, Population Division; 2015. https://esa.un.org/unpd/wpp/publications/files/key_findings_wpp_2015.pdf. Accessed 12 Dec 2016.

[10] Peltzer KF, Mbelle N, Makiwane M, Tabane C, Phaswana-Mafuya N, Zuma K, et al. Study on global AGEing and adult health (SAGE), wave 1: South Africa National Report. 2012. http://www.hsrc.ac.za/en/research-outputs/view/5903. Accessed 2 Dec 2016.

[11] Peltzer K, Phaswana-Mafuya N. Patient experiences and health system responsiveness among older adults in South Africa. Glob Health Action. 2012;5. 10.3402/gha.v5i0.18545. 10.3402/gha.v5i0.18545 PMC3509423 23195515

[12] Ameh S, Gómez-Olivé FX, Kahn K, Tollman SM, Klipstein-Grobusch K. Predictors of health care use by adults 50 years and over in a rural south African setting. Glob Health Action. 2014;7. 10.3402/gha.v7.24771. 10.3402/gha.v7.24771 PMC4119936 25087686

[13] Ameh S, Klipstein-Grobusch K, D’ambruoso L, Kahn K, Tollman SM, Gómez-Olivé FX (2016). Quality of integrated chronic disease care in rural South Africa: user and provider perspectives. Health Policy Plan.

[14] Harris B, Eyles J, Penn-Kekana L, Thomas L, Goudge J (2014). Adverse or acceptable: negotiating access to a post-apartheid health care contract. Glob Health.

[15] Hasumi T, Jacobsen KH (2014). Healthcare service problems reported in a national survey of south Africans. Int J Qual Health Care.

[16] Lopes Ibanez-Gonzalez D, Norris SA. Chronic non-communicable disease and healthcare access in middle-aged and older women living in Soweto, South Africa. PLoS ONE. 2013;8. 10.1371/journal.pone.0078800. 10.1371/journal.pone.0078800 PMC3812146 24205316

[17] Phaswana-Mafuya N, Peltzer K, Chirinda W, Kose Z, Hoosain E, Ramlagan S, et al. Self-rated health and associated factors among older south Africans: evidence from the study on global ageing and adult health. Glob Health Action. 2013;6. 10.3402/gha.v6i0.19880. 10.3402/gha.v6i0.19880 PMC3567200 28140909

[18] Lehohla P. Census 2011: profile of older persons in South Africa. Pretoria: Statistics South Africa; 2014. http://www.statssa.gov.za/publications/Report-03-01-60/Report-03-01-602011.pdf. Accessed 6 Dec 2016.

[19] Charasse-Pouélé C, Fournier M (2006). Health disparities between racial groups in South Africa: a decomposition analysis. Soc Sci Med.

[20] Coovadia H, Jewkes R, Barron P, Sanders D, McIntyre D (2009). The health and health system of South Africa: historical roots of current public health challenges. Lancet.

[21] Statistics South Africa (2017). Vulnerable groups series II: the social profile of older persons, 2011–2015.

[22] Gerber AM, Botes R, Mostert A, Vorster A, Buskens E (2016). A cohort study of elderly people in Bloemfontein, South Africa, to determine health-related quality of life and functional abilities. South Afr Med J Suid-Afr Tydskr Vir Geneeskd.

[23] Gómez-Olivé FX, Thorogood M, Bocquier P, Mee P, Kahn K, Berkman L (2014). Social conditions and disability related to the mortality of older people in rural South Africa. Int J Epidemiol.

[24] Kalula SZ, Ferreira M, Swingler GH, Badri M. Risk factors for falls in older adults in a south African Urban Community. BMC Geriatr. 2016;16. 10.1186/s12877-016-0212-7. 10.1186/s12877-016-0212-7 PMC4766747 26912129

[25] Robert SA, Cherepanov D, Palta M, Dunham NC, Feeny D, Fryback DG (2009). Socioeconomic status and age variations in health-related quality of life: results from the National Health Measurement Study. J Gerontol B Psychol Sci Soc Sci.

[26] Ryvicker M, Gallo WT, Fahs MC (2012). Environmental factors associated with primary care access among urban older adults. Soc Sci Med.

[27] Schatz E, Gómez-Olivé X, Ralston M, Menken J, Tollman S (2012). The impact of pensions on health and wellbeing in rural South Africa: does gender matter?. Soc Sci Med.

[28] Yamada T, Chen C-C, Murata C, Hirai H, Ojima T, Kondo K (2015). Access disparity and health inequality of the elderly: unmet needs and delayed healthcare. Int J Environ Res Public Health.

[29] Joubert J, Bradshaw D, Steyn K, Fourie J, Temple N (2006). Population ageing and health challenges in South Africa. Chronic diseases of lifestyle in South Africa: 1995–2005.

[30] Pillay NK, Maharaj P, Maharaj P (2013). Population ageing in Africa. Aging and health in Africa.

[31] Clarke LH, Bennett EV, Korotchenko A (2014). Negotiating vulnerabilities: how older adults with multiple chronic conditions interact with physicians. Can J Aging.

[32] Jacinto AF, Dozzi Brucki SM, Porto CS, de Arruda MM, de Albuquerque CV, Nitrini R (2014). Suggested instruments for general practitioners in countries with low schooling to screen for cognitive impairment in the elderly. Int Psychogeriatr.

[33] Jacinto AF, Brucki S, Porto CS, Martins M, De A, Nitrini R (2011). Detection of cognitive impairment in the elderly by general internists in Brazil. Clinics..

[34] Kalula S, Petros G. Responses to dementia in less developed countries with a focus on SA. IFA Glob Ageing 2011;11. http://www.ifa-fiv.org/wp-content/uploads/global-ageing/7.1/7.1.kalula.petros.pdf. Accessed 5 Nov 2016.

[35] Lopes Ibanez-Gonzalez D, Mendenhall E, Norris SA (2014). A mixed methods exploration of patterns of healthcare utilization of urban women with non-communicable disease in South Africa. BMC Health Serv Res.

[36] Scheffler E, Visagie S, Schneider M. The impact of health service variables on healthcare access in a low resourced urban setting in the Western cape, South Africa. Afr J Prim Health Care Fam Med. 2015;7. 10.4102/phcfm.v7i1.820. 10.4102/phcfm.v7i1.820 PMC4656938 26245611

[37] Statistics South Africa (2016). General household survey 2016.

[38] Strauss AL, Corbin J. Basics of qualitative research: grounded theory procedures and techniques. CA: Sage: Thousand Oaks; 1990.

[39] Glaser BG, Strauss AL (1967). The discovery of grounded theory: strategies for qualitative research.

[40] Charmaz K (2006). Constructing grounded theory: a practical guide through qualitative analysis.

[41] Kitzinger J (1994). The methodology of focus groups: the importance of interaction between research participants. Sociol Health Illn.

[42] Morgan DL, Spanish MT (1984). Focus groups: a new tool for qualitative research. Qual Sociol.

[43] Altheide D, Johnson J, Denzin N, Lincoln Y (2011). Reflections on interpretive adequacy in qualitative research. The SAGE handbook of qualitative research.

[44] Draper CA, Draper CE, Bresick GF (2014). Alignment between chronic disease policy and practice: case study at a primary care facility. PLoS One.

[45] Ameh SS. Chronic disease care in primary health care facilities in rural south African settings. Thesis. 2016. http://wiredspace.wits.ac.za/handle/10539/22244. Accessed 24 May 2017.

[46] Sokhela DG, Sibiya MN, Gwele NS (2016). Experiences of health care providers in the fast queue service point in primary health care facilities in Ethekwini district, South Africa. Afr J Nurs Midwifery.

[47] Gaede BM, Gaede B. Doctors as street-level bureaucrats in a rural hospital in South Africa. Rural Remote Health. 2016;16 http://www.rrh.org.au/Articles/printviewnew.asp?ArticleID=3461. Accessed 5 Nov 2016. 26851960

[48] Sirsawy U, Steinberg W, Raubenheimer J (2016). Levels of burnout among registrars and medical officers working at Bloemfontein public healthcare facilities in 2013. South Afr Fam Pract..

[49] Labonté R, Sanders D, Mathole T, Crush J, Chikanda A, Dambisya Y (2015). Health worker migration from South Africa: causes, consequences and policy responses. Hum Resour Health.

[50] Rossouw L, Seedat S, Emsley RA, Suliman S, Hagemeister D (2013). The prevalence of burnout and depression in medical doctors working in the Cape Town metropolitan municipality community healthcare clinics and district hospitals of the provincial government of the Western cape: a cross-sectional study. South Afr Fam Pract.

[51] Gross K, Pfeiffer C, Obrist B (2012). “Workhood”-a useful concept for the analysis of health workers’ resources? An evaluation from Tanzania. BMC Health Serv Res.

[52] Jewkes R, Abrahams N, Mvo Z (1998). Why do nurses abuse patients? Reflections from south African obstetric services. Soc Sci Med.

[53] Tibandebage P, Mackintosh M (2005). The market shaping of charges, trust and abuse: health care transactions in Tanzania. Soc Sci Med.

[54] Ferreira M, Lindgren P (2008). Elder abuse and neglect in South Africa: a case of marginalization, disrespect, exploitation and violence. J Elder Abuse Negl.

[55] Kinkel HF, Adelekan AM, Marcus TS, Wolvaardt G (2012). Assessment of service quality of public antiretroviral treatment (ART) clinics in South Africa: a cross-sectional study. BMC Health Serv Res.

[56] Kelly G (2017). Patient agency and contested notions of disability in social assistance applications in South Africa. Soc Sci Med.

[57] South African Health Review 2017. Durban: Health Systems Trust. http://www.hst.org.za/publications/south-african-health-review-2017. Accessed 5 Oct 2018.

[58] Institute of Medicine (2001). Crossing the quality chasm: a new health system for the 21st century.

[59] Starfield B (2009). Primary care and equity in health: the importance to effectiveness and equity of responsiveness to peoples’ needs. Humanity Soc.

[60] Levinson W, Lesser CS, Epstein RM (2010). Developing Physician Communication Skills For Patient-Centered Care. Health Aff (Millwood).

[61] Starfield B (2011). Is patient-centered care the same as person-focused care?. Perm J.

[62] Haskard Zolnierek KB, DiMatteo MR (2009). Physician communication and patient adherence to treatment: a meta-analysis. Med Care.

[63] Walker L, Gilson L (2004). ‘We are bitter but we are satisfied’: nurses as street-level bureaucrats in South Africa. Soc Sci Med.

[64] Le Marcis F, Grard J (2015). Ethnography of everyday ethics in a South African medical Ward. Real governance and practical norms in sub-Saharan Africa: the game of the rules.

[65] Fassin D (2008). The elementary forms of care. Soc Sci Med.

[66] Alharbi TSJ, Carlström E, Ekman I, Jarneborn A, Olsson L-E (2014). Experiences of person-centred care-patients’ perceptions: qualitative study. BMC Nurs.

[67] Bray P, Cummings DM, Morrissey S, Thompson D, Holbert D, Wilson K (2013). Improved outcomes in diabetes care for rural African Americans. Ann Fam Med.

